# Novel mechanisms of endothelial dysfunction in diabetes

**DOI:** 10.4103/0975-3583.64432

**Published:** 2010

**Authors:** Guang Yang, Rudolf Lucas, Ruth Caldwell, Lin Yao, Maritza J. Romero, Robert W. Caldwell

**Affiliations:** 1 *Department of Vascular Biology Center, Medical College of Georgia, Augusta, GA, USA*; 2 *Department of Pharmacology and Toxicology, Medical College of Georgia, Augusta, GA, USA*

**Keywords:** Arginase, diabetes, endothelial dysfunction, protein kinase C, reactive oxygen species, tumor necrosis factor

## Abstract

Diabetes mellitus is a major risk factor for cardiovascular morbidity and mortality. This condition increases the risk of developing coronary, cerebrovascular, and peripheral arterial disease fourfold. Endothelial dysfunction is a major contributor to the pathogenesis of vascular disease in diabetes mellitus patients and has recently received increased attention. In this review article, some recent developments that could improve the knowledge of diabetes-induced endothelial dysfunction are discussed.

## INTRODUCTION

According to the World Health Organization, diabetes mellitus now affects about 220 million people worldwide,[1] and the growth in its prevalence represents a global health crisis already accounting for more than 10% of the total healthcare expenditure in many countries.[2] In the USA, over 24 million children and adults (almost 8% of the entire population) have diabetes, whereas another 57 million have prediabetes and are thus likely to develop the disease unless they make lifestyle changes.[3] Diabetes without proper treatment can cause many complications, with cardiovascular diseases accounting for up to 80% of premature mortality.[4]

The endothelium, once considered a mere selectively permeable barrier between the bloodstream and the outer vascular wall, is now recognized to be a crucial homeostatic organ, fundamental for the regulation of the vascular tone and structure.[5] Therefore, endothelial dysfunction during diabetes has been associated with a number of pathophysiologic processes.[6] A considerable body of evidence in humans indicates that endothelial dysfunction is closely associated with the development of diabetic retinopathy,[7] nephropathy, and atherosclerosis in both type 1 and type 2 diabetes.[8] In this article, the recent findings on the mechanisms of endothelial dysfunction in diabetes, which could contribute to the development of new treatment options are discussed.

## PHYSIOLOGIC ROLE OF THE VASCULAR ENDOTHELIUM

The healthy endothelial monolayer is optimally positioned in order to respond to physical and chemical signals, by producing a wide range of factors that regulate vascular tone, cellular adhesion, thromboresistance, smooth muscle cell proliferation, and vessel wall inflammation. The importance of the endothelium was first recognized by its effect in limiting the vascular tone.[9] The vascular endothelium also regulates blood flow and, limits leukocyte adhesion and platelet aggregation by producing nitric oxide (NO), prostacyclin, and ectonucleotidases. As such, inflammatory activity in the vessel wall is blunted. In addition, the endothelium regulates vascular permeability to nutrients, macromolecules, and leukocytes; limits activation of the coagulation cascade by the thrombomodulin/protein C, heparin sulfate/antithrombin, and tissue factor/tissue factor pathway inhibitor interactions; and regulates fibrinolysis by producing tissue activator of plasminogen (t-PA) and its inhibitor, PAI-1.[10]

## DIABETES-INDUCED ENDOTHELIAL DYSFUNCTION

The term endothelial dysfunction refers to a condition in which the endothelium loses its physiologic properties and shifts toward a vasoconstrictor, prothrombotic, and proinflammatory state.[11] Endothelial dysfunction has been associated with a variety of processes, including hypertension, atherosclerosis, aging, heart and renal failure, coronary syndrome, obesity, vasculitis, infections, sepsis, rheumatoid arthritis, thrombosis, smoking as well as with type 1 and type 2 diabetes [6]

Diabetes mellitus, often simply referred to as diabetes, is a condition with elevated blood glucose levels, as a result of either the body not producing enough insulin, or because cells do not properly respond to the insulin that is produced. The latter condition is characterized by fasting hyperglycemia and by a high risk of atherothrombotic disorders affecting the coronary, cerebral, and peripheral arterial trees.[12] Despite improvement in the management of patients with unstable coronary syndromes, diabetes is still linked to a substantial increase in mortality and morbidity among these patients.[13] Accordingly, developing new therapies for the treatment of diabetic patients is of great clinical importance.[14,15]

In diabetes, dysfunction of the vascular endothelium is regarded as an important factor in the pathogenesis of diabetic micro-and macroangiopathy.[8] There are three main sources contributing to endothelial dysfunction in diabetes: (1) hyperglycemia and its immediate biochemical sequelae directly alter endothelial function; (2) high glucose (HG), which influences endothelial cell functioning indirectly by the synthesis of growth factors and vasoactive agents in other cells and alters endothelial monolayer permeability; and (3) the components of the metabolic syndrome that can affect endothelial function.[8]

There are many signaling molecules involved in the pathogenesis of endothelial dysfunction. In the following paragraphs, recent studies on this topic, mainly focusing on the roles of arginase and reactive oxygen species (ROS), protein kinase C (PKC), and tumor necrosis factor (TNF), are addressed.

## ROLE OF ARGINASE AND REACTIVE OXYGEN SPECIES IN DIABETES-ASSOCIATED ENDOTHELIAL DYSFUNCTION

Conditions contributing to diabetic vascular remodeling and dysfunction include the effects of oxidative stress and decreased NO bioavailability.[16‐19] NO production by endothelial NOS (eNOS) is critically involved in maintaining the integrity and stability of the vascular endothelium, preventing platelet aggregation and leukocyte adhesion, and maintaining blood flow.[20] Availability of the semi-essential amino acid L-arginine is required for eNOS activity and NO production and is therefore essential for vascular integrity and function. Arginase is a hydrolytic enzyme, which converts L-arginine into urea and ornithine and exists as 2 isoforms: arginase I and II.[21] Whereas arginase I is a cytosolic enzyme, expressed at high levels in the liver, arginase II is a mitochondrial enzyme expressed primarily in the extrahepatic tissues, especially in the kidney. Knockdown of arginase I has been shown to restore NO signaling in the vasculature of old rats.[22] Both arginase I and II have been found in endothelial cells, arginase I being the dominant isoform.[23]

Arginase and eNOS compete for their common substrate, L-arginine. As such, increased arginase activity can lead to eNOS dysfunction.[23,24] We have shown that hepatic and vascular arginase activity is increased in diabetic rats and that arginase I expression and activity are increased in aortic endothelial cells exposed to HG.[24] TNF has also been shown to induce arginase activity.[25] Furthermore, arginase actions causing endothelial dysfunction, as indicated by decreased NO availability, are blocked by the Rho kinase inhibitor Y-27632.[26] Additionally, an inhibitor of arginase reversed diabetes-induced endothelial dysfunction in the coronary vessels of diabetic rats.[24] Also, arginase was found to mediate retinal inflammation in lipopolysaccharide (LPS)-induced uveitis.[21]

**Figure 1:**
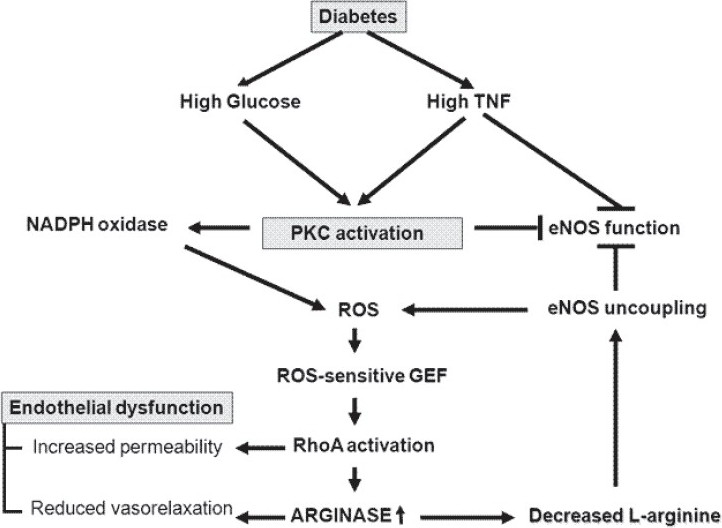
Mechanisms leading to endothelial dysfunction in diabetes

Taken together, these findings[21‐26] suggest that arginase and RhoA may be mediators of diabetes-induced inflammatory effects in vascular disease. Apart from arginase, ROS also play an important role in vascular dysfunction in diabetes, although the source of their generation remains elusive. Overproduction of superoxide can lead to scavenging of NO and to its reduced bioavailability.[27,28] ROS have been implicated in increased arginase activity and expression. Indeed, arginase activation can cause uncoupling of eNOS by reducing the supply of L-arginine. The uncoupled eNOS uses molecular oxygen to produce superoxide, thereby further reducing NO and increasing ROS formation [Figure 1].

## IMPLICATION OF PROTEIN KINASE C

An important glucose-induced alteration in cellular metabolism that may account for endothelial dysfunction is activation of PKC. Hyperglycemia causes *de novo* synthesis of diacylglycerol, leading to the activation of PKC, a pathway now demonstrated in all vascular tissues involved in diabetic complications.[29] Of interest, the adverse effects of elevated glucose levels on acetylcholine-induced relaxation of rabbit aorta and rat pial arterioles were restored by the addition of PKC-inhibitors.[30,31] Diabetes-induced translocation of PKC-alpha to renal membranes was associated with increased nicotinamide adenine dinucleotide phosphate oxidase-dependent superoxide generation.[32] It has been proposed that HG concentrations rather specifically activate the beta II isoform of PKC.[33] However, the PKC alpha isoform, which is activated by HG in bovine aortic endothelial cells, has also been suggested to play an important role in diabetes mellitus-associated endothelial dysfunction, since specific antisense or pharmacologic inhibition completely abolished the effects of HG on endothelial cell permeability.[34] The reported activity of PKC-alpha on endothelial permeability is at least partially mediated by inducing phosphorylation of p115RhoGEF,[35] a guanine nucleotide exchange factor (GEF) for Rho GTPase.[36] Because active RhoA is implicated in arginase induction,[24] it suggests that PKC-alpha might also be involved in regulation of arginase activity.

## TUMOR NECROSIS FACTOR: A MOONLIGHTING CYTOKINE IN ENDOTHELIAL DYSFUNCTION

Human TNF is a 51-kDa homotrimeric protein. TNF is generated as a membrane-bound precursor that is cleaved by the metalloproteinase family member TNF-alpha converting enzyme, giving rise to the soluble protein.[37] The main sources of the cytokine are activated macrophages and T cells. TNF binds to 2 different TNF receptors, TNF-R1 (55 kDa) and TNF-R2 (75 kDa), at least one of which is expressed in most somatic cells.[37] Soluble TNF has the highest affinity for TNF-R1, whereas membrane-bound TNF preferentially interacts with TNF-R2.[38] Apart from the ligand TNF, also the receptors exist as membrane-associated and soluble forms.[37] TNF-R1, but not TNF-R2, contains a death domain, which signals apoptosis upon the formation of the death-inducing signaling complex[37] Although not carrying a death domain, TNF-R2 has nevertheless been implicated in apoptosis regulation in microvascular endothelial cells.[39]

Spatially distinct from its receptor binding sites, TNF carries a lectin-like domain, recognizing specific oligosaccharides, such as *N, N*'-diacetylchitobiose and branched trimannoses,[40] which can be mimicked by the 17-amino acid circular TIP peptide (amino acid sequence: CGQRETPEGAEAKPWYC).[41] Three residues, namely, T105, E107, and E110, appear to be crucial for this activity. The TIP peptide exerts a lytic activity toward bloodstream forms of African trypanosomes,[41] which occurs upon binding to the oligosaccharides expressed in the variant-specific glycoprotein of the parasites. More importantly, the TIP peptide also increases sodium transport in lung microvascular endothelial cells.[42] Interestingly, the activities of the lectin-like domain of TNF cannot be inhibited by the soluble TNF receptors.[41]

TNF is one of the key inflammatory mediators that is expressed during a variety of inflammatory conditions and initiates the expression of an entire spectrum of inflammatory cytokines ranging from many interleukins to interferons.[43] It is suggested that inflammation is an effector of not only endothelial dysfunction, but also insulin resistance and atherosclerosis.[44] Under inflammatory conditions, TNF can increase the expressions of adhesion molecules, such as vascular cell adhesion molecule (VCAM-1) and intercellular adhesion molecule (ICAM-1); and as such promote the adherence of monocytes.[45] Moreover, TNF can affect NO production by decreasing eNOS expression[46] and increase the production of ROS in neutrophils and endothelial cells through NAPH oxidase,[47] xanthine oxidase,[48] and uncoupled NOS.[49] The pivotal role of TNF in diabetes-induced endothelial dysfunction can also be manifested by the observation that endothelial function is close to normal in a TNF-knockout diabetic mouse model.[50]

The generation of TNF is increased during diabetes, and the cytokine has been shown to upregulate the expression of arginase in endothelial cells, which leads to endothelial dysfunction during ischemia reperfusion injury in mice.[25] Recent studies have indicated that TNF can affect endothelial barrier integrity.[51] by means of (1) inducing apoptosis of lung microvascular endothelial cells,[39] which can contribute to the disruption of the endothelial barrier during acute lung injury and acute respiratory distress syndrome;[52] (2) by inducing the production of ROS;[53] and (3) by directly increasing endothelial permeability in a RhoA/ROCK-dependent manner.[54] PKC-alpha activation was proposed to be involved in TNF-mediated increases in permeability of pulmonary microvessel endothelial monolayers.[55] On the other hand, the lectin-like domain of TNF, mimicked by the TIP peptide, can increase endothelial monolayer resistance in the presence of bacterial toxins, by means of inhibiting listeriolysin-induced PKC-alpha activation, which in turn inhibits RhoA activation and myosin light chain phosphorylation.[56] Moreover, the lectin-like domain of TNF can reduce ischemia-reperfusion–induced ROS generation in a lung transplantation model[57] As such, the lectin-like domain of TNF can potentially oppose the deleterious receptor-mediated activities of the cytokine on the endothelium.[58]

## CONCLUSION

The present communication has reviewed some recent studies on diabetes-induced endothelial dysfunction and has discussed the important roles of arginase, PKC and TNF in this complicated pathological condition. The interactions between these molecules and the proposed mechanism of diabetes-induced endothelial dysfunction are summarized in Figure 1. New insights into these mechanisms and into crucial targets of endothelial dysfunction in diabetes may lead to novel strategies for treatment in the future.
